# Protocol Across study: longitudinal transdiagnostic cognitive functioning, psychiatric symptoms, and biological parameters in patients with a psychiatric disorder

**DOI:** 10.1186/s12888-020-02624-x

**Published:** 2020-05-11

**Authors:** Dorien H. Nieman, UnYoung Chavez-Baldini, Nienke C. Vulink, Dirk J. A. Smit, Guido van Wingen, Pelle de Koning, Arjen L. Sutterland, Roel J. T. Mocking, Claudi Bockting, Karin J. H. Verweij, Anja Lok, Damiaan Denys

**Affiliations:** Department of Psychiatry, Amsterdam University Medical Centers (location AMC), University of Amsterdam, Meibergdreef 5, 1105 AZ Amsterdam, Netherlands

**Keywords:** Study protocol, Transdiagnostic, Cognitive functioning, Psychiatric disorders

## Abstract

**Background:**

Patients with psychiatric disorders, such as major depressive disorder, schizophrenia or obsessive-compulsive disorder, often suffer from cognitive dysfunction. The nature of these dysfunctions and their relation with clinical symptoms and biological parameters is not yet clear. Traditionally, cognitive dysfunction is studied in patients with specific psychiatric disorders, disregarding the fact that cognitive deficits are shared across disorders. The Across study aims to investigate cognitive functioning and its relation with psychiatric symptoms and biological parameters transdiagnostically and longitudinally.

**Methods:**

The study recruits patients diagnosed with a variety of psychiatric disorders and has a longitudinal cohort design with an assessment at baseline and at one-year follow-up. The primary outcome measure is cognitive functioning. The secondary outcome measures include clinical symptoms, electroencephalographic, genetic and blood markers (e.g., fatty acids), and hair cortisol concentration levels.

**Discussion:**

The Across study provides an opportunity for a transdiagnostic, bottom-up, data-driven approach of investigating cognition in relation to symptoms and biological parameters longitudinally in patients with psychiatric disorders. The study may help to find new clusters of symptoms, biological markers, and cognitive dysfunctions that have better prognostic value than the current diagnostic categories. Furthermore, increased insight into the relationship among cognitive deficits, biological parameters, and psychiatric symptoms can lead to new treatment possibilities.

**Trial registration:**

Netherlands Trial Register (NTR): NL8170.

## Background

Patients with psychiatric disorders often have cognitive deficits [1]. These deficits have been associated with psychosocial dysfunction in a variety of disorders, including depression [2, 3], schizophrenia [4], and bipolar disorder [5]. Cognition encompasses a number of interrelated mental activities, such as attention, learning, memory, problem-solving, and planning [1], all of which are important for daily life functioning. In fact, cognitive dysfunctions may form an important underlying factor between psychiatric symptoms and functional outcomes [6, 7]. For instance, patients with schizophrenia have expressed a particular desire to treat cognitive deficits above the amelioration of their psychotic symptoms in order to function in daily life [8]. Cognitive deficits can also have an impact on other dimensions of psychiatric disorders by potentially contributing to and exacerbating cognitive biases [9]. However, cognitive dysfunction continues to be ineffectively treated because evidence-based treatments for cognitive dysfunction are scarce.

Previous research into cognitive dysfunction in psychiatric patients was mainly conducted in patient populations within specific diagnostic categories. However, high rates of comorbidity and heterogeneity are present across and within disorders [10–12]. The heterogeneity within diagnostic categories and overlap of diagnostic criteria between disorders can be demonstrated by the fact that there are 227 ways to meet the criteria for major depressive disorder due to the polythetic definition of the disorder [13], and that at least half of patients with depressive disorder have a comorbid anxiety disorder [14, 15]. Heterogeneity in and comorbidity across disorders manifest not only at the symptom level but also in behavior, physiology, and cognitive functioning. This could be a factor in lack of consensus regarding neuropsychological profiles for psychiatric disorders.

In addition, whether cognitive dysfunctions are generalized (i.e., global cognitive deficit) or more specific (i.e., psychotic disorders are associated with impairment in cognitive flexibility) is not yet clear. A reason why this may be difficult to determine is that studies often employ a limited assessment of cognition. Cognition is a multifaceted construct and consists of multiple domains, and some cognitive domains have sub-domains [1]. For instance, executive functioning consists of different abilities, such as cognitive flexibility, verbal fluency, and strategy use, while it is often assessed with one test [16]. Memory encompasses immediate and delayed memory, and includes different mechanisms, such as retrieval and consolidation [1]. The use of single assessments to measure such complex processes may give a limited view on cognition, corroborating the need for multiple tests that assess specific cognitive domains.

Additionally, there is a need for further investigation into which domains of cognition are trait- or state-dependent. Cognitive deficits that persist after remission suggest that certain cognitive domains may be trait-dependent. For instance, a review of cognitive functioning in young adults with major depressive disorder suggests that executive functioning and cognitive control deficits persist despite remission of clinical symptoms whereas other cognitive domains seem to be more dependent on clinical status [17]. Nonetheless, findings tend to be mixed and many studies investigated only one domain or are cross-sectional, so longitudinal studies with various cognitive domain assessments are necessary to assess any possible changes in functioning. Longitudinal investigations could elucidate whether certain domains of cognitive dysfunction are related to clinical state or whether they reflect, for instance, abnormal neurodevelopment and genetic vulnerabilities [18]. Furthermore, insight into potential causal relationships could be gained with a longitudinal approach, such as whether psychiatric symptoms or biological measurement outcomes impact cognitive functioning at a later time or vice versa.

Furthermore, biological mechanisms related to changes in cognition are not yet well-established. Inclusion of biological parameters may provide further insight into pathophysiological mechanisms associated with cognitive deficits and phenotypic expressions of disorders. For instance, cortisol awakening response is associated with memory deficits in patients with psychotic disorders [19] and medicated patients with major depressive disorder [20]. In addition, cognitive functioning shows a relationship with electroencephalogram (EEG) derivatives, such as the P300 and mismatch negativity (MMN) event-related potential, in individuals diagnosed with a variety of psychiatric disorders [21–23] and in healthy subjects [24, 25]. Other physiological parameters are also associated with cognition and psychopathology, such as inflammatory markers, which show an association with poor performance on memory, language, and attention tests in women with post-traumatic stress disorder [26]. Recently, the role of polyunsaturated fatty acids (PUFAs) has also been garnering attention, and PUFA deficits have been transdiagnostically associated with diverse psychiatric disorders and cognition [27–30]. However, there has been a lack in solid findings regarding biomarkers associated with cognitive functioning in psychiatric disorders. This may once again be because studies analyze biological parameters focusing on specific psychiatric disorders, most often excluding those with comorbidity and thus possibly disregarding heterogeneity or subgroups within disorders. The inclusion of blood markers, EEG and cortisol as biological parameters in our study was informed by recent evidence of their transdiagnostic relationship with psychopathology [31–38].

Therefore, a transdiagnostic approach may be optimal to study the general role of cognition in psychiatric disorders. A transdiagnostic approach acknowledges heterogeneity and comorbidity of symptoms because it does not view mental disorders as categorically distinct entities. As cognitive dysfunction occurs in patients with a variety of psychiatric disorders [1], it should be treated as a transdiagnostic dimension [39]. Supporting this, the Research Domain Criteria (RDoC) framework also regards cognition as a transdiagnostic domain [40]. A transdiagnostic approach therefore provides an opportunity for a bottom-up data-driven method of investigating cognition in relation to symptoms and biological parameters that is not bound to diagnostic categories.

### Objectives

The objectives of the ongoing Across study are to: 1) investigate cognitive dysfunctions transdiagnostically across different psychiatric disorders, 2) link cognitive dysfunctions with psychiatric symptoms and biological parameters, and 3) investigate the longitudinal course of cognitive dysfunctions in relation to symptoms and biological parameters.

Due to the diverse measures included in this study, a wide-range of research questions can be investigated. Some hypotheses to be tested include: 1) executive functioning is impaired in psychiatric patients, 2) cortisol levels are associated with memory functioning, 3) lower concentrations of omega-3 PUFAs are associated with poorer cognitive functioning, and 4) verbal memory dysfunction persist despite improvements in psychiatric symptoms. Other research questions can be investigated with the acquired data.

The findings of the Across study could elucidate relationships among cognition, psychiatric symptoms, and biomarkers. This could lead to insight into mechanisms related to cognitive dysfunctions, which can be used as targets in treatment. Furthermore, longitudinal assessments can provide information on state and trait components of cognitive dysfunction.

## Methods/design

### Study design and procedure

The Across study is an ongoing, naturalistic longitudinal cohort study and consists of the assessment of cognitive performance, psychiatric symptoms, and collection of biological data (DOI 10.17605/OSF.IO/YHVTB) [41]. All patients visiting the Department of Psychiatry at the Amsterdam University Medical Centers (Amsterdam UMC), location Academic Medical Center (AMC) for an intake are referred to the study and can choose which parts to participate in after they received information about the study and provide informed consent. All testing and data collection takes place at the Amsterdam UMC.

After intake, blood is drawn at a laboratory for the assessment of blood markers. On a later date, cognitive performance is assessed using a computerized battery, which is followed by the completion of self-report questionnaires on various symptom domains. This takes 2 to 3 h to complete. After completion of the questionnaires, a hair sample is collected to measure cortisol levels, and 45 min of EEG recordings is obtained, which provides data on information-processing deficits. Underage participants (ages 14–17) complete a shorter version of the questionnaires and do not partake in the EEG data collection as EEG parameters are known to develop strongly during these periods [42, 43]. Participants are invited to be assessed again 1 year later if they consent to being contacted. The follow-up included the exact same measures, except for the blood donation.

The study protocol was approved by the Medical Ethical Review Committee and the Biobank Review Committee of the Amsterdam UMC (ABR no. NL55751.018.15). Biological data and material are stored in the Amsterdam UMC Biobank, a secured facility established specifically for the storage and management of biological materials. Patients are assigned a study number and patient data are stored securely to ensure confidentiality. All researchers undergo thorough training and receive extensive supervision to ensure quality of data collection.

### Study population

The study population consists of individuals between the ages of 14 to 75 years who have a diagnosis of at least one psychiatric disorder. Participants are recruited through the tertiary care Department of Psychiatry at the Amsterdam UMC, location AMC, the Netherlands, if they meet the participation criteria (Table 1).

**Table 1:** Criteria for inclusion, exclusion, and discontinuation of participation

**Inclusion criteria**	
1. Ability to give informed consent	
2. DSM-IV-TR axis I or DSM-5 diagnosis	
3. Aged 14–75 years at intake	
4. For under-aged participants, consent will also be obtained from the participant’s parents in addition to the participant’s consent	
5. Fluent in Dutch	
6. Clinically stable	
**Exclusion criteria**	
1. High risk of suicide	
2. Unstable medical disorder	
3. Premorbid IQ < 70	
4. History of a clinically significant abnormality of the neurological system (including dementia and other cognitive disorders or significant head injury) or any history of seizure (excluding febrile seizure)	
**Discontinuation criteria**	
1. Voluntary discontinuation by the patient who is at any time free to discontinue his or her participation in the study, without consequences to further treatment	
2. Safety reasons as judged by the investigator	
3. Severe non-compliance to the protocol as judged by the investigator	
4. Incorrect enrolment (i.e., the patient did not meet or does no longer meet the required inclusion criteria) of the patient	
5. Patient lost to follow-up due to no response	
6. Development of exclusion criteria	

All patients receive a Diagnostic and Statistical Manual of Mental Disorders (DSM)-IV-TR or DSM-5 diagnosis [44, 45] from a trained psychiatrist within the Amsterdam UMC. As of September 2019, 1091 patients completed a baseline assessment and 272 patients completed a one-year follow-up assessment. On average, 14 patients agreed to participate in the Across study per month and the study will continue the coming years.

The current sample has a mean (SD) premorbid IQ of 100.09 (13.3) and 86.5% of the sample were of Caucasian ethnicity. On average, the sample consisted of middle-aged adults with a mean (SD) age of 34.6 (14.1) years, and there was nearly an equal number of males and females (47.2 and 52.8%, respectively). The most common diagnoses are: disruptive, impulse-control, and conduct disorders (*n* = 409), schizophrenia spectrum and other psychotic disorders (*n* = 191), obsessive-compulsive spectrum disorders (*n* = 164), depressive disorders (*n* = 122), and anxiety disorders (*n* = 64).

### Measures

Demographic information, such as gender, ethnicity, age, and education, is obtained through self-report.

#### Primary outcome measures

The primary outcome measure is cognitive functioning, which is assessed with the Cambridge Neuropsychological Test Automated Battery (CANTAB [46]). The CANTAB test battery is composed of the following subtests: Motor screening (MTS), Verbal Recognition Memory (VRM), Rapid Visual Information Processing (RVP), Intra/ Extradimensional Set Shift (IED), Choice reaction time (CRT), One Touch Stockings of Cambridge (OTS), Paired Associates Learning (PAL), Graded Naming Test (GNT), and Spatial Working Memory (SWM). Descriptions of the subtests can be found in Table 2.

**Table 2:** Description of Cambridge Neuropsychological Test Automated Battery subtests

Subtests	Description
Verbal Recognition Memory (VRM)	Assesses free recall, and immediate and delayed recognition memory for verbal information
Rapid Visual Information Processing (RVP)	Tests visual sustained attention and processing speed
Intra/ Extradimensional Set Shift (IED)	Assesses rule acquisition and attentional set shifting
Choice reaction time (CRT)	Measures alertness and motor speed
One Touch Stockings of Cambridge (OTS)	A planning test which gives a measure of frontal lobe functioning
Paired Associates Learning (PAL)	Assesses visual episodic memory and learning
Spatial Working Memory (SWM)	Assesses working memory and strategy use

In addition to CANTAB, the following tests are administered: the Dutch National Adult Reading Test (NART) to assess premorbid IQ [47]; the California Verbal Learning Test to measure episodic verbal learning and memory [48]; and the semantic Verbal Fluency of the Groninger Intelligence Test [49].

#### Secondary outcome measures

These measures include psychometrically established self-report questionnaires on dimensions of psychopathology.

Substance use-related disorders are assessed with two questionnaires. The Alcohol Use Disorder Identification Test (AUDIT) consists of 10 items that assess alcohol consumption, drinking behaviors, and alcohol-related problems on scale of 0–4 [50] with a median internal reliability of Cronbach alpha in the 0.80s across numerous studies [51]. Cannabis use problems are screened with the Cannabis Use Disorder Identification Test (CUDIT), which consists of 10 items measuring frequency and dependence with a positive predictive power of 84.6% and sensitivity of 73.3% at a cut-off of 8 [52]. In addition, participants are asked about the frequency of use for numerous substances, including coffee, cigarettes, stimulants (e.g., amphetamines), sedatives (e.g., barbiturates), opiates (e.g., heroin), and others.

The Prodromal Questionnaire (PQ-16) assesses the occurrence and severity of At Risk Mental State symptoms for a first psychosis with 2 items on negative symptoms, 5 items on unusual thought content/delusional ideas/paranoia, and 9 items on perceptual abnormalities/hallucinations [53]. A previous study found a Cronbach’s alpha for the total score of 0.77 and all item-total correlations of at least 0.31 [53].

The Yale-Brown Obsessive Compulsive Scale (Y-BOCS) measures the severity and type of obsessive-compulsive symptoms with 10 items [54]. Item-total correlations were at least 0.36 with a mean Cronbach’s alpha of 0.89 for internal consistency [54].

Anxiety-related symptoms are measured with two questionnaires. The Hamilton Anxiety Scale (HAM-A) assesses the severity of somatic, cognitive, and affective symptoms of anxiety with 13 items [47] and demonstrates good interrater reliability [55]. Anxiety in social interactions and fear of scrutiny by others is assessed with the 20-item Social Interaction Anxiety Scale (SIAS [56]). The SIAS demonstrates high levels of internal consistency (α = 0.94), test-retest reliability at 12 weeks (*r* = 0.92), and sensitivity to change with treatment [56].

The severity of depressive symptoms is measured with Inventory of Depressive Symptomatology Self-Report (IDS-SR) with 30 items pertaining to mood, cognition, arousal, suicidality, and sleep [57]. The IDS demonstrates good internal consistency (α = 0.85) and is applicable to different types of depression [58].

Self-esteem in relation to social contact, achievements, and competency is assessed with the Self-esteem Rating Scale- Short Form (SERS-SR [59]). The SERS-SR demonstrates good test-retest reliability for the positive scale (*r* = 0.90) and the negative scale (r = 0.91) and high internal consistency for each scale (respectively, α = 0.91 and α = 0.87).

The Impact of Events Scale-Revised (IES-R) assesses subjective distress caused by traumatic events with 22 items and is composed of three subscales: avoidance, intrusions, and hyperarousal [60]. The IES-R shows good internal consistency (α = 0.96) and a cut-off score of 33 provided a sensitivity of 0.91, a specificity of 0.82, positive predictive power of 0.90, and negative predictive power of 0.84 [60].

The Work and Social Adjustment Scale (WSAS) is a 5-item questionnaire that measures general impairment in different domains of daily life, including work, social activities, and leisure activities [61]. The WSAS is sensitive to disorder severity and treatment-related changes and demonstrated a test-retest correlation of 0.73 and internal consistency ranging from α = 0.70 to 0.94 [61].

The Psychiatric Dimensions Questionnaire was developed at the Amsterdam UMC and contains 26 items, which assess a variety of transdiagnostic concepts such as identity, autonomy, and self-control, that are commonly affected in patients with a psychiatric disorder.

Anhedonia is measured with the Anhedonia Scale [62], in which participants rate 21 items related to pleasure from physical activity, hearing, seeing, touching, tasting, sex, and smelling.

The AUDIT, CUDIT, IES-R,Y-BOCS, WSAS, and Anhedonia Scale are administered only to adult patients. These self-report questionnaires are administered on a computer with the Computer Diagnostic Leiden (CDL) program [63]. Most tests and questionnaires include norms and cut-off scores that can aid in interpreting the range of performance.

Patients are also asked about drug and medication use and their experience of participating in the study. Other information, such as the diagnosis and family history, is also collected during the clinical intake.

#### Biological measures

##### Blood markers

22 ml non-fasting blood samples are collected at baseline on the day of the intake at a laboratory within the Amsterdam UMC as part of standard blood collection for clinical purposes. Blood samples are stored in five tubes: 1) PAXgene for RNA; 2) EDTA 6 ml for DNA; 3) EDTA 4 ml for red blood cells, white blood cells, and platelets; 4) lithium heparin for plasma determinations (e.g., cholesterol and hormones); and 5) serum for antibodies and other proteins. After collection, the blood samples are stored in − 80 °C cryostorage at the Amsterdam UMC Biobank. The appropriate pre-processing steps (e.g., genotyping for DNA) will be conducted in order to analyze various blood markers, such as cytokines, DNA/RNA, and fatty acids. The methods to analyze fatty acids have been described previously in Mocking, Assies [64]; in brief, erythrocytes are first separated, washed and frozen. Subsequently, fatty acid concentrations are analyzed using capillary gas chromatography and expressed in pmol/10^6^ erythrocyte.

##### Electroencephalogram (EEG)

EEG is assessed with a WaveGuard cap with Ag/AgCl electrodes with standard 10/10 layout fed into the 64-channel ANT TMSI Refa amplifier, using Fpz as ground, horizontal EOG electrodes affixed to the outer canthus and vertical EOG electrodes affixed above and below the right eye, two mastoid channels (M1/M2). The vertex electrode (Cz) is used as the recording reference. The resting state EEG and auditory oddball task are recorded in a session of 45 min. Recordings were sampled at 512 Hz with a 128 Hz high-pass filter. Eyes-closed resting state recordings take 5 min, eyes-opened resting state recordings take 3 min, and the auditory oddball task takes about 12 min. During the auditory oddball task, patients watch a nature documentary film as they listen to a series of beeps and press a button whenever there is a high-pitched beep. EEG data are collected only from adult patients.

##### Hair cortisol

A string of about 100 hairs is cut from the posterior vertex region of the scalp [65] to assess cortisol levels over the course of months in which 1 cm of hair is approximately equal to 1 month of mean cortisol levels [66, 67]. Hair samples are stored at room temperature at the Amsterdam UMC Biobank. To assess confounders, patients complete a questionnaire regarding hair-related characteristics, such as hair coloring, frequency of hair washing per week, use of hair products, and use of corticosteroids.

### Power calculation

The power calculation was focused on the detection of a bivariate correlation between two quantitative measures as it will determine subsequent data reduction techniques. The proposed sample sizes are shown in Table 3, which would allow 80% power to detect a correlation of at least 0.08 at various alpha levels while accounting for an attrition rate of 20%. The necessary sample size could differ according to the number of tests and the minimum correlation we want to detect.

**Table 3:** Power calculation: Detected two-tailed bivariate correlation at 80% power

Correlation coefficient	Alpha	N^a^
0.08	0.0005	3492
0.14	0.0005	1134
0.2	0.0005	550
0.08	0.001	3192
0.014	0.001	1037
0.2	0.001	503
0.08	0.01	2184
0.14	0.01	709
0.2	0.01	345

^a^ Sample size accounting for a 20% attrition rate

### Proposed statistical analyses

The association between cognitive dysfunctions, symptoms, and biological parameters will be analyzed. To elucidate models that most adequately explain the correlational patterns between variables, data reduction techniques will be employed (e.g., factor analyses or network analyses) as determined by the structure of the correlational matrices. Other data reduction techniques, such as (graph) clustering analyses can be used, to determine whether symptom clusters match diagnosis.

Linear regression analyses will be conducted with cognition scores as the outcome measure and dummy coded diagnostic groups as the predictors to investigate cognitive deficits across different psychiatric disorders. Age, gender, education, ethnicity, and medication use will be included as covariates as deemed appropriate. Similar models will be built for the biological outcomes variables.

The longitudinal course of cognitive dysfunctions will be investigated with repeated measures analyses or a regression model using cognition change scores as the outcome variable and baseline cognition scores as predictor. Other predictors of changes will be investigated by conducting regression analyses with cognition change scores as the outcome measure and biological parameters and clinical symptoms as predictors with the baseline cognition scores as a covariate. Additionally, prediction of cognitive functioning and symptom course can be analyzed using machine learning. Age, gender, education, ethnicity, and medication use will also be included as covariates if deemed appropriate. If there are doubts about bias due to age-related effects, we will conduct sensitivity analyses.

Statistical analyses will be performed in IBM SPSS Statistics 24 [68] or R [69].

## Discussion

The objectives, study population characteristics, and assessment methods of the Across study are presented here to provide a detailed methodological reference for future Across papers. The aim of the study is to investigate cognitive functioning and its relation with symptoms and biological parameters transdiagnostically and longitudinally.

The Across study has a number of strengths. Firstly, the team of researchers and clinicians involved is multidisciplinary and has academic expertise in the specific topics of this study. Patients are seen during intakes by professionals with extensive clinical experience, and the research is set up and led by principal investigators with relevant expertise in genetics, EEG, and cognition research. Furthermore, the longitudinal design, large sample size, and transdiagnostic biopsychosocial approach add value to this study. The latter is especially important as it allows for a more comprehensive understanding of cognition in relation to biological parameters and psychiatric symptoms across disorders. In addition, the study utilizes a variety of instruments, providing researchers the opportunity to investigate different aspects of cognition and psychiatric disorders. Cognition is also assessed as a complex and multifaceted construct with the use of tasks focusing on cognitive domains (and sub-domains). This also adds to the comprehensive nature of the study as cognition can be rather complex due to the numerous factors that interact on a variety of levels. Further, a relatively large transdiagnostic sample increases the ecological validity of this study as it includes minor and adult patients with various and comorbid psychiatric disorders, allowing for a better reflection of clinical reality. Moreover, there are relatively few exclusion criteria, and the inclusion criteria are broad, adding to the generalizability of the sample. Lastly, this study is performed at one institute, ensuring a more homogeneous approach than multicenter studies, which often encounter difficulty with merging data across centers and ensuring assessors are trained uniformly.

The study results should be interpreted with a few methodological considerations in mind. First, there may be a selection bias as the patients who are willing to participate may differ from patients who refuse. This is inherent to psychiatric research, and in order to obtain the most representative sample possible, lenient inclusion and exclusion criteria were used. Second, the quality of assessments may be influenced by the large number of research assistants involved, despite the investment in proper training. Quality checks are put into place to ensure that the protocol is being followed and that assistants carry out the assessments correctly. Third, the patients included in the study tend to have severe psychiatric disorders because the Amsterdam UMC is an institution that provides tertiary care based on specific referrals. This may reduce the generalizability of the results to less severely affected patients, and there may be both over- and under-representations of certain disorders. Findings may nonetheless still be representative for institutions similar to the Amsterdam UMC. Fourth, while a few hypotheses have already been determined, the vast dataset allows for the possibility of many other future research questions. Re-using datasets is a cost-effective and time saving way of doing research but on the other hand it leads to limitations in predefining the analysis plan. However, most biobank studies combine hypothesis-driven research with more general data-collection and data-driven analyses.

Although research into cognition is not necessarily sparse, much remains uncertain and unknown about the nature of cognitive dysfunctions in psychiatric patients. For example, a transdiagnostic construct such as anhedonia could be related to cognitive dysfunction. Cognitive dysfunctions remain undertreated as a result of the limited knowledge regarding the nature of cognitive deficits, despite its high occurrence in and heavy burden for psychiatric patients. The Across study may find new clusters of symptoms, biological markers, and cognitive dysfunctions that have better prognostic value than the current diagnostic categories. Furthermore, increased insight into the relationship among cognitive deficits, biological parameters, and psychiatric symptoms can lead to new treatment possibilities.

## Data Availability

The raw data can be requested from dr. Dorien Nieman, email: d.h.nieman@amsterdamumc.nl. Data requests will be discussed in the Across Executive board. The raw data are not publicly available due to their clinical and confidential nature.

## Abbreviations

EEG: Electroencephalogram
MMN: Mismatch negativity
PUFAs: Polyunsaturated fatty acids
RDoC: Research Domain Criteria
Amsterdam UMC: Amsterdam University Medical Centers
AMC: Academic Medical Center
DSM: Diagnostic and Statistical Manual of Mental Disorders
CANTAB: Cambridge Neuropsychological Test Automated Battery
MTS: Motor screening
VRM: Verbal Recognition Memory
RVP: Rapid Visual Information Processing
IED: Intra/ Extradimensional Set Shift
CRT: Choice reaction time
OTS: One Touch Stockings of Cambridge
PAL: Paired Associates Learning
GNT: Graded Naming Test
SWM: Spatial Working Memory
NART: Dutch National Adult Reading Test
AUDIT: Alcohol Use Disorder Identification Test
CUDIT: Cannabis Use Disorder Identification Test
PQ-16: Prodromal Questionnaire
Y-BOCS: Yale-Brown Obsessive Compulsive Scale
HAM-A: Hamilton Anxiety Scale
SIAS: Social Interaction Anxiety Scale
IDS-SR: Inventory of Depressive Symptomatology Self-Report
SERS-SR: Self-esteem Rating Scale- Short Form
IES-R: Impact of Events Scale-Revised
WSAS: Work and Social Adjustment Scale
CDL: Computer Diagnostic Leiden
EDTA: Ethylenediaminetetraacetic acid
DNA: Deoxyribonucleic acid
RNA: Ribonucleic acid
